# Parasympathetic Effect Induces Cell Cycle Activation in Upper Limbs of Paraplegic Patients with Spinal Cord Injury

**DOI:** 10.3390/ijms20235982

**Published:** 2019-11-27

**Authors:** Ahreum Baek, Ji Cheol Shin, Min-Young Lee, Sung Hoon Kim, Jiyong Kim, Sung-Rae Cho

**Affiliations:** 1 Department and Research Institute of Rehabilitation Medicine, Yonsei University College of Medicine, Seoul 03722, Korea; ahreumbaek@yonsei.ac.kr (A.B.); jcsevrm@yuhs.ac (J.C.S.); alsdud8099@hanmail.net (M.-Y.L.); 2 Department of Rehabilitation Medicine, Yonsei University Wonju College of Medicine, Wonju 26426, Korea; kimrehab@yonsei.ac.kr; 3 Department of Physical Medicine and Rehabilitation, Inje University Ilsanpaik Hospital, 170 Juhwa-ro, Ilsanseo-gu, Goyang 10380, Korea; 4 Brain Korea 21 PLUS Project for Medical Science, Yonsei University College of Medicine, Seoul 03722, Korea; 5 Yonsei Stem Cell Center, Avison Biomedical Research Center, Yonsei University College of Medicine, Seoul 03722, Korea; 6 Rehabilitation Institute of Neuromuscular Disease, Yonsei University College of Medicine, Seoul 03722, Korea

**Keywords:** cell cycle, cell proliferation, spinal cord injury, parasympathetic effect

## Abstract

The present study aimed to investigate gene expression changes related to cell cycle activation in patients with spinal cord injury (SCI) and to further evaluate the difference between the upper and lower limbs of SCI patients. Fibroblasts were obtained from the upper and lower limbs of SCI patients and healthy subjects. To investigate gene expression profiling in the fibroblasts from SCI patients compared to the healthy subjects, RNA-Seq transcriptome analysis was performed. To validate the parasympathetic effects on cell cycle activation, fibroblasts from upper or lower limbs of SCI patients were treated with the anticholinergic agents tiotropium or acetylcholine, and quantitative RT-PCR and Western blot were conducted. Cell proliferation was significantly increased in the upper limbs of SCI patients compared with the lower limbs of SCI patients and healthy subjects. The pathway and genes involved in cell cycle were identified by RNA-Seq transcriptome analysis. Expression of cell-cycle-related genes *CCNB1*, *CCNB2*, *PLK1*, *BUB1*, and *CDC20* were significantly higher in the upper limbs of SCI patients compared with the lower limbs of SCI patients and healthy subjects. When the fibroblasts were treated with tiotropium the upper limbs and acetylcholine in the lower limbs, the expression of cell-cycle-related genes and cell proliferation were significantly modulated. This study provided the insight that cell proliferation and cell cycle activation were observed to be significantly increased in the upper limbs of SCI patients via the parasympathetic effect.

## 1. Introduction

Spinal cord injury (SCI) initiates several primary and secondary mechanisms causing neuronal cell death, autonomic and immune dysfunction, sustained neurological deficits, and a significantly high risk of morbidity and mortality [1,2]. Following a primary insult such as compression or contusion of the spinal cord, secondary injury mechanisms, including apoptosis, loss of myelination, and abnormal ionic homeostasis, lead to the spread of damage from the initial site of injury [3,4].

The cell cycle is crucial in normal physiological conditions; thus, its dysfunction leads to aberrant cell proliferation [5]. Cell-cycle-related genes and proteins that are upregulated immediately following SCI are associated with induced glial scar formation and chronic inflammation [6] and induce activation of both astroglia and microglia as well as the proliferation of these cells [7,8,9]. According to those effects, functional recovery and microglia-induced inflammatory responses were found to be significantly improved following the administration of a cell-cycle-inhibiting drug in a rat model of SCI [10,11]. It has also been reported that cell cycle inhibitors may be potential treatment measures for aged mouse models of SCI [12]. Taken together, activation of the cell cycle contributes to the pathophysiology of SCI [6,7,8,9,13], but inhibition of the cell cycle should also be neuroprotective in SCI [10,11,12]. 

However, most studies have been conducted using in vivo animal models of SCI, while few studies have provided confirmation of the identified genes in human tissues. Therefore, to identify differentially expressed genes (DEGs) involved in patients with SCI, we performed gene expression profiling of fibroblasts from patients with SCI by RNA-Seq transcriptome analysis.

## 2. Results

### 2.1. Characteristics of SCI Patients and Healthy Subjects 

To obtain fibroblasts, dermal punch biopsies were taken from three paraplegic patients with SCI and three healthy subjects. In the healthy subjects, fibroblasts were obtained from the chin, back, and buttock. In the patients with SCI, fibroblasts were obtained from two different regions: the deltoid muscle of the nondominant arm and the quadriceps muscle. Clinical information of the patients with SCI is presented in Table S1. Healthy subjects were all males, aged 32, 63, and 76 years old. Similarly, the patients with SCI were all males, aged 57, 49, and 59 years old. The mean ages of healthy subjects and patients with SCI were 57 and 55 years, respectively. Mean disease duration in patients with SCI was 48.7 months. All of the patients were in chronic stage post-SCI, had fully recovered from spinal shock status, and upper motor signs were predominantly expressed. The neurological level of injury of the patients with SCI were T5, T4, and T3 and all patients lacked supraspinal modulation of sympathetic neurons. Their upper limb muscle (represented by the deltoid muscle) was located above the neurological level of injury, while their lower limb muscle (represented by the quadriceps muscle) was located below the neurological level of injury. The American Spinal Injury Association Impairment Scale [14] for all patients was A (indicating complete SCI). The “completeness” of SCI was confirmed by neurological examination and somatosensory evoked potential test. 

### 2.2. Evaluation of Cell Proliferation in SCI Patients and Healthy Subjects 

**Figure 1:**
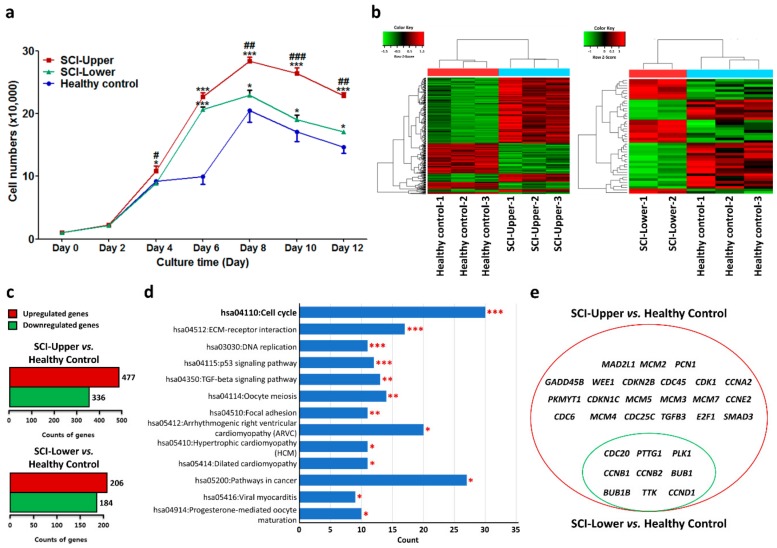
Gene expression profile by transcriptome analysis in spinal cord injury (SCI) patients and healthy subjects. (**a**) Line graphs show cell numbers of fibroblasts from SCI patients and healthy subjects from day 0 to 12 at passage 4. Blue line represents fibroblasts from healthy subjects (indicated as healthy control, *n* = 9), red line represents fibroblasts from deltoid muscle (indicated as SCI-Upper, *n* = 9), and green line represents fibroblasts from quadriceps muscle (indicated as SCI-Lower, *n* = 6). * *p* < 0.05 and *** *p* < 0.001 comparison with healthy control, and ^#^ *p* < 0.05, ^##^ *p* < 0.01, and ^###^ *p* < 0.001 comparison with the SCI-Lower from one-way analysis of variance followed by Bonferroni post hoc test. (**b**) Heat map of differentially expressed genes in the fibroblasts from SCI-Upper (*n* = 3) compared to healthy control (*n* = 3) (left panel) and in the fibroblasts from SCI-Lower (*n* = 2) compared to healthy control (right panel). The two-way hierarchical clustering method was used to normalize the value, and the relative expression level of the samples is indicated by color key and z-score. High expression levels are represented as red and low levels are represented as green. (**c**) Bar graphs show the number of differentially expressed genes with fold change ≥ |2.0| in the fibroblasts from SCI-Upper compared to healthy control (upper graph) and from SCI-Lower compared to healthy control (lower graph). Red bar represents upregulated genes and green bar represents downregulated genes. (**d**) Kyoto Encyclopedia of Genes and Genomes pathway analyses of the differentially expressed genes in the fibroblasts from SCI-Upper compared to healthy control. Significant terms (* *p* < 0.05, ** *p* < 0.01, and *** *p* < 0.001) are highlighted in red. (**e**) The Venn diagrams show the differentially expressed genes for the cell cycle pathway between SCI-Upper compared to healthy control (represented as red circle) and SCI-Lower compared to healthy control (represented as green circle).

To evaluate cell proliferation, fibroblasts were seeded in six-well plates and counted with an ADAM automatic cell counter on days 2, 4, 6, 8, 10, and 12 (Figure 1a). At passage 4, the cell proliferation in the upper limbs of SCI patients was significantly increased from day 4 compared with healthy control and lower limbs of SCI patients, respectively. The cell proliferation in the lower limbs of SCI patients was also significantly increased compared with healthy control from day 6. The cell number in the upper and lower limbs of SCI patients was particularly higher than the healthy control at passage 4 (Figure 1a). Especially, the cell number in the upper limbs of SCI patients was significantly higher than the other two groups. These results indicate that cell proliferation was significantly increased in the upper limbs of SCI patients.

### 2.3. Analysis of the Differentially Expressed Genes in SCI Patients and Healthy Subjects 

Next, a transcriptome array was performed to identify DEGs in the upper limbs of SCI patients, lower limbs of SCI patients, and healthy control at passage 4. A heat map of mRNA expression representing transcripts in the upper limbs of SCI patients compared to healthy control is shown in Figure 1b (left panel) and that in the lower limbs of SCI patients compared to healthy control is shown in Figure 1b (right panel).

In the upper limbs of SCI patients compared to healthy control, 15,572 genes were differentially expressed. Among those genes, 477 transcripts were 2-fold higher and 336 transcripts were 2-fold lower in the upper limbs of SCI patients compared with healthy control (Figure 1c, upper panel). In the lower limbs of SCI patients compared to healthy control, 15,732 genes were differentially expressed. Among those genes, 206 transcripts were 2-fold higher and 184 transcripts were 2-fold lower in the upper limbs of SCI patients compared with healthy control (Figure 1c, lower panel). 

Especially, DEGs in the SCI patients compared to healthy control were classified with enriched Kyoto Encyclopedia of Genes and Genomes pathways using DAVID software (Table 1 and Table 2). Among these pathways, the cell cycle pathway was significantly enriched in both the upper (Figure 1d) and lower limbs of SCI patients compared with healthy control (*p* < 0.05). Additionally, nine shared common DEGs, such as *cell division cycle 20 (CDC20)*, *pituitary tumor transforming gene 1 (PTTG1)*, *polo-like kinase 1 (PLK1)*, *cyclin B2 (CCNB2)*, *cyclin B1 (CCNB1)*, *BUB1 mitotic checkpoint serine/threonine kinase B (BUB1B)*, *BUB1 mitotic checkpoint serine/threonine kinase (BUB1)*, *monopolar spindle 1 kinase (TTK)*, and *cyclin D1 (CCND1)*, were significantly upregulated both in the upper and lower limbs of SCI patients compared with healthy control (Figure 1e, left panel). The fold change (FC) ratio for those genes is shown in Table 3.

### 2.4. Validation of Differential Gene Expression in SCI Patients and Healthy Subjects 

**Figure 2:**
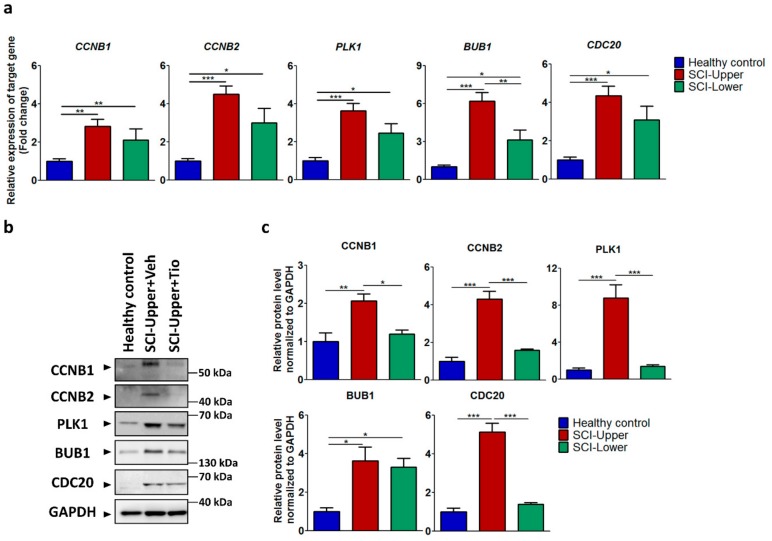
Validation of identified genes by transcriptome analysis in SCI patients and healthy subjects. (**a**) The relative expression of target genes for qRT-PCR was calculated using the 2^−ΔΔCt^ method. Blue bar represents fibroblasts from healthy subjects (indicated as healthy control, *n* = 12), red bar represents fibroblasts from deltoid muscle (indicated as SCI-Upper, *n* = 12), and green bar represents fibroblasts from quadriceps muscle (indicated as SCI-Lower, *n* = 8). * *p* < 0.05, ** *p* < 0.01, and *** *p* < 0.001 from one-way analysis of variance followed by Bonferroni post hoc test. (**b**) Western blot analysis was performed using antibodies against CCNB1, CCNB2, PLK1, BUB1, CDC20, and GAPDH. (**c**) Comparison of relative protein expression from the SCI-Upper (*n* = 9) and SCI-Lower (*n* = 9) versus the healthy control (*n* = 6). * *p* < 0.05, ** *p* < 0.01, and *** *p* < 0.001 from one-way analysis of variance followed by Bonferroni post hoc test.

Among nine shared common genes, expression levels of *CCNB1*, *CCNB2*, *PLK1*, *BUB1*, and *CDC20* were validated by qRT-PCR in the upper and lower limbs of SCI patients compared to healthy control (Figure 2a). The gene expression ratios are presented in Table S2. In the upper limbs of SCI patients compared with healthy control, *CCNB1* (*p* < 0.01), *CCNB2* (*p* < 0.001), *PLK1* (*p* < 0.001), *BUB1* (*p* < 0.001), and *CDC20* (*p* < 0.001) were significantly increased. In the lower limbs of SCI patients compared with healthy control, *CCNB1* (*p* < 0.01), *CCNB2* (*p* < 0.05), *PLK1* (*p* < 0.05), *BUB1*, and *CDC20* (*p* < 0.05) were increased. In particular, *BUB1* (*p* < 0.01) was significantly increased in the upper limbs of SCI patients compared with the lower limbs of SCI patients (Figure 2a).

Next, the protein expression of CCNB1, CCNB2, PLK1, BUB1, and CDC20 were validated by Western blot in the upper and lower limbs of SCI patients compared to healthy control (Figure 2b). The protein expression ratios are presented in Table S3. In the upper limbs of SCI patients compared with healthy control, CCNB1 (*p* < 0.01), CCNB2 (*p* < 0.001), PLK1 (*p* < 0.001), BUB1 (*p* < 0.01), and CDC20 (*p* < 0.001) were significantly increased (Figure 2c). In the lower limbs of SCI patients compared with healthy control, CCNB1, CCNB2, PLK1, BUB1 (*p* < 0.05), and CDC20 were increased. In particular, CCNB1 (*p* < 0.05), CCNB2 (*p* < 0.001), PLK1 (*p* < 0.001), and CDC20 (*p* < 0.001) were significantly increased in the upper limbs of SCI patients compared with the lower limbs of SCI patients (Figure 2c). These results demonstrate that the cell cycle was significantly activated in the upper limbs of SCI patients.

### 2.5. Validation of Differential Gene Expression after Tiotropium and Acetylcholine Treatment

The anticholinergic agents tiotropium and acetylcholine have been reported to regulate the parasympathetic nervous system [15,16]. Therefore, tiotropium or distilled water were treated in fibroblasts from SCI-Upper and acetylcholine or distilled water were treated in fibroblasts from SCI-Lower to validate the correlation between patients with SCI and parasympathetic effect. 

**Figure 3:**
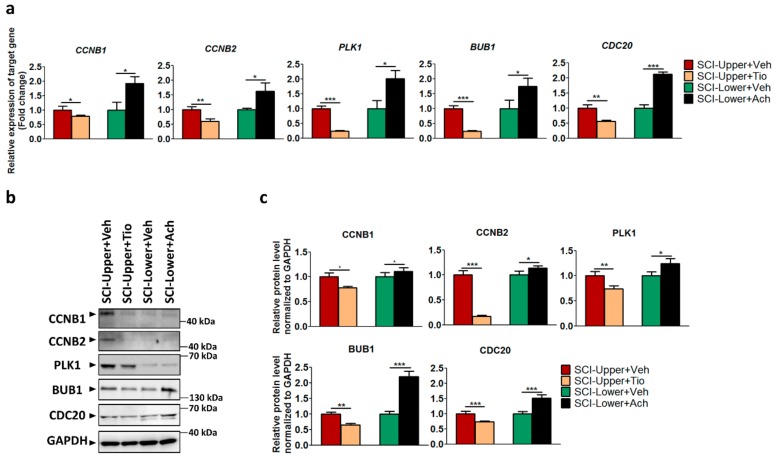
The effects of tiotropium and acetylcholine in SCI patients. (**a**) The relative expression of target genes for qRT-PCR was calculated using the 2^−ΔΔCt^ method. Red bar represents fibroblasts from SCI-Upper+treated with vehicle (indicated as SCI-Upper+Veh, *n* = 7–8), light brown bar represents fibroblasts from SCI-Upper treated with tiotropium (indicated as SCI-Upper+Tio, *n* = 6), green bar represents fibroblasts from SCI-Lower treated with vehicle (indicated as SCI-Lower+Veh, *n* = 5–6), and black bar represents fibroblasts from SCI-Lower treated with acetylcholine (SCI-Lower+Ach, *n* = 5–6). * *p* < 0.05, ** *p* < 0.01, and *** *p* < 0.001 from independent *t*-test. (**b**) Western blot analysis was performed using antibodies against CCNB1, CCNB2, PLK1, BUB1, CDC20, and GAPDH. (**c**) Comparison of relative protein expression from SCI-Upper+Tio versus SCI-Upper+Veh and SCI-Lower+Ach versus SCI-Lower+Veh. * *p* < 0.05, ** *p* < 0.01, and *** *p* < 0.001 from independent *t*-test; *n* = 8 per group.

In the upper limbs of SCI patients treated with tiotropium (indicated as the SCI-Upper+Tio group), the expression level of *CCNB1* (*p* < 0.05), *CCNB2* (*p* < 0.01), *PLK1* (*p* < 0.001), *BUB1* (*p* < 0.001), and *CDC20* (*p* < 0.01) was significantly decreased compared with the upper limbs of SCI patients treated with distilled water (indicated as the SCI-Upper+Veh group), as revealed by qRT-PCR (Figure 3a). However, in the lower limbs of SCI patients treated with acetylcholine (indicated as the SCI-Lower+Ach group), the expression level of *CCNB1* (*p* < 0.05), *CCNB2* (*p* < 0.05), *PLK1* (*p* < 0.05), *BUB1* (*p* < 0.05), and *CDC20* (*p* < 0.001) was significantly increased compared with the lower limbs of SCI patients treated with distilled water (indicated as the SCI-Lower+Veh group). The gene expression ratios are presented in Table S4.

Next, the protein expression of CCNB1, CCNB2, PLK1, BUB1, and CDC20 was confirmed by Western blot (Figure 3b,c). In the SCI-Upper+Tio group compared with the SCI-Upper+Veh group, the expression of CCNB1 (*p* < 0.05), CCNB2 (*p* < 0.001), PLK1 (*p* < 0.01), BUB1 (*p* < 0.01), and CDC20 (*p* < 0.001) was significantly decreased. However, in the SCI-Lower+Ach group compared with the SCI-Lower+Veh group, the expression of CCNB1 (*p* < 0.05), CCNB2 (*p* < 0.05), PLK1 (*p* < 0.05), BUB1 (*p* < 0.001), and CDC20 (*p* < 0.001) was significantly increased. The protein expression ratios are presented in Table S5. These results suggest that cell cycle activation was modulated via the parasympathetic effect in patients with SCI.

### 2.6. Evaluation of Cell Proliferation after Tiotropium and Acetylcholine Treatment

**Figure 4:**
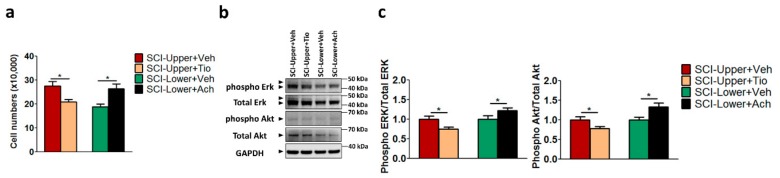
The effects of tiotropium and acetylcholine on cell proliferation in in SCI patients. (**a**) Bar graphs show cell number after treatment with tiotropium and acetylcholine in the fibroblasts from patients with SCI. Red bar represents fibroblasts from SCI-Upper treated with vehicle (indicated as SCI-Upper+Veh), light brown bar represents fibroblasts from SCI-Upper treated with tiotropium (indicated as SCI-Upper+Tio), green bar represents fibroblasts from SCI-Lower treated with vehicle (indicated as SCI-Lower+Veh), and black bar represents fibroblasts from SCI-Lower treated with acetylcholine (SCI-Lower+Ach). * *p* < 0.05 from independent *t*-test. (**b**) Western blot analysis was performed using antibodies against phosphorylated Erk, Total Erk, phosphorylated Akt, Total Akt, and GAPDH. (**c**) Comparison of relative protein expression from SCI-Upper+Tio versus SCI-Upper+Veh and SCI-Lower+Ach versus SCI-Lower+Veh. * *p* < 0.05 from independent *t*-test; *n* = 8 per group.

To validate the correlation between cell proliferation and parasympathetic effect in patients with SCI, cell proliferation was evaluated in the SCI fibroblast after anticholinergic agent or vehicle treatment (Figure 4a) and the cell numbers of each group were as follows: SCI-Upper+Veh group (27.53 × 10^4^), SCI-Upper+Tio group (20.86 × 10^4^), SCI-Lower+Veh group (18.79 × 10^4^), and SCI-Lower +Ach group (26.33 × 10^4^). The cell number of the SCI-Upper+Tio group was significantly decreased compared with the SCI-Upper+Veh group (*p* < 0.05). Conversely, the cell number of fibroblasts from the SCI-Lower+Ach group was significantly increased compared with the SCI-Lower+Veh group (*p* < 0.05).

Furthermore, we investigated the ERK and AKT pathways, which have been reported to play an important role in the regulation of growth and proliferation [17] by Western blot analysis (Figure 4b,c). The expression of phosphorylated Erk/Total Erk and phosphorylated Akt/Total Akt was significantly decreased in the SCI-Upper+Tio group compared with the SCI-Upper+Veh group (*p* < 0.05 and *p* < 0.05, respectively). However, the expression of phosphorylated Erk/Total Erk and phosphorylated Akt/Total Akt was significantly increased in the SCI-Lower+Ach group compared with the SCI-Lower+Veh group (*p* < 0.05 and *p* < 0.05, respectively). The protein expression ratios are presented in Table S6. These results suggest that cell proliferation was modulated via the parasympathetic effect in patients with SCI.

## 3. Discussion

In the present study, increased cell proliferation and cell cycle activation were shown in the upper limbs of paraplegic patients with SCI, suggesting that the parasympathetic nervous system affected cell proliferation and the cell cycle above the neurological level of injury rather than below it. The possible cause of the difference is the influence of the autonomic nervous system. Most spinal cord lesions at or above the sixth thoracic (T6) spinal cord segment result in autonomic dysreflexia [18,19]. Whereas sympathetic hyperactivity is triggered by autonomic dysreflexia, resulting in systemic vasoconstriction below the level of the spinal cord lesion [20], parasympathetic activity is induced above the level of the lesion [21,22]. In this study, each patient had spinal cord lesions above the T6 level with pure supraspinal disinhibition. In such cases, the parasympathetic nervous system is relatively dominant in the upper limbs above the neurological level of injury, while the sympathetic nervous system is relatively dominant in the lower limbs below the neurological level of injury [23]. Previous studies have shown that the parasympathetic nervous system affects cell proliferation. Pieper et al. reported that acetylcholine, the major neurotransmitter of the parasympathetic nervous system, stimulates the proliferation of human lung fibroblasts and treatment with antimuscarinic drugs inhibits acetylcholine-induced proliferation [15]. Oben et al. revealed that acetylcholine stimulates proliferation and collagen gene expression of myofibroblastic hepatic stellate cells [24]. Kurzen et al. reported that the proliferation of dermal fibroblasts is affected by acetylcholine activity [25].

In the current study, we postulated that parasympathetic dominance above the neurological level of injury may influence cell proliferation. To confirm this, further experiments were conducted using acetylcholine and tiotropium treatments. When fibroblasts from the lower limbs of SCI patients were treated with acetylcholine, which is the final product released by the parasympathetic nervous system [16], cell proliferation and the expression of target genes increased in the acetylcholine-treated fibroblasts. On the other hand, when fibroblasts from upper limbs of SCI patients were treated with tiotropium, which is a competitive antagonist for acetylcholine [15], cell proliferation and the expression of target genes were reduced in the tiotropium-treated fibroblasts. These results confirmed the relationship between parasympathetic activity, cell proliferation, and cell-cycle-related gene expression.

In this study, the upregulated genes, such as *CCNB1*, *CCNB2*, *PLK1*, *BUB1*, and *CDC20*, were all involved in maintaining chromosomal stability. Overexpression of these genes is associated with tumorigenesis by increasing chromosomal instability. Activation of the cell cycle pathway after SCI and subsequent cell proliferation is associated with a wide range of damage caused by secondary mechanisms after SCI and may also be associated with a higher risk of cancer in patients with SCI [26]. 

Another possible cause for the difference in gene expression in the upper and lower extremities of patients with SCI is the effect of physical activity. Lee et al. and Chen et al. both reported that physical activity increases astrocyte proliferation, which is associated with regeneration of capillaries in rat models of ischemic brain injury [27,28]. Szczodry et al. reported that myofibroblast expression in the patellar tendon in rats was increased in a treadmill exercise group compared with the control group [29]. In the current study, all of the patients who participated had complete paraplegia and were able to voluntarily exercise their upper extremities but were unable to spontaneously contract their lower extremities. Although voluntary activity of the lower extremities was not possible, therapeutic application of neuromuscular electrical stimulation may result in exercise effects by contracting muscles [30]. 

So far, no studies have been reported on the relationship between physical activity and cell proliferation after SCI. However, considering the results of previous studies, it is possible that the differences in the amount of physical activity in the upper and lower extremities of the patients may have contributed to the differences in gene expression associated with cell proliferation and cell cycle in the current study.

As for the limitations of this study, our data are restricted to three SCI individuals and three healthy volunteers on a small scale, and the ages of the healthy subjects in this study were not evenly distributed. A previous study has shown that age is associated with the regulation of mitotic genes [31]. Therefore, further studies should be conducted on a large scale and considered under a comparison group containing similar ages. Moreover, the direct comparison between the upper- and lower-limb-derived cells of healthy and SCI patients from the same region should be investigated further.

In addition, we hypothesized that SCI causes changes in the autonomic nervous system throughout the whole body. The patient-derived fibroblasts, which were readily available, were used for our experiment since the biopsy of neuronal tissues is invasive and technically difficult. It will be further needed to establish human-induced pluripotent stem cells (hiPSCs), generating neural lineage cells differentiated from the hiPSCs to clarify the relationship between the autonomic nervous system and either above or below the neurological level of injury in the patients with SCI. Based on this in vitro study, we should further apply these experimental strategies to SCI patients as in vivo studies to evaluate the neuroprotection effects of anticholinergic medicines.

Taken together, cell proliferation was significantly increased in the upper limbs of SCI patients compared with the lower limbs of SCI patients and healthy subjects. Furthermore, the cell cycle pathway was also significantly activated resulting from the parasympathetic nervous system being predominant in the upper limbs of SCI patients compared with the lower limbs of SCI patients. Our study may provide the relationship between cell cycle and parasympathetic effect in SCI patients; thus, these findings will be applied in further cell therapy strategies regarding the inhibition of cell cycle, antiproliferation properties, and the neuroprotective effect in SCI patients.

## 4. Materials and Methods

### 4.1. Subjects

Patients and healthy subjects signed informed consent forms prior to the study. Consent for the collection of human dermal fibroblast samples was included in the study’s written informed consent form. The Institutional Review Board of Severance Hospital, Yonsei University Health System approved the consent procedure as well as the entire study (No. 4-2012-0028).

### 4.2. Preparation of Fibroblast Cells 

We performed 3 mm punch biopsies through the full thickness of the dermis in the upper and lower limbs of the SCI patients with confirmed SCI by a dermatology specialist. Dermal fibroblasts were also obtained from healthy subjects in the same way. Biopsy samples were transferred to a culture dish with growth media, Dulbecco’s modified Eagle’s medium containing 10% fetal bovine serum and 1% penicillin/streptomycin, and incubated in a humidified 5% CO_2_ atmosphere at 37 °C. Tiotropium, (SigmaAldrich, St. Louis, MO, USA) and acetylcholine (SigmaAldrich, St. Louis, MO, USA) were treated in the fibroblasts from SCI-Upper and SCI-Lower, respectively. The concentration and treatment conditions followed previously established methods [15].

### 4.3. Analysis of Cell Proliferation 

To analyze fibroblast proliferation, 10,000 cells were seeded in six-well plates with growth media. The number of cells per plate were counted with an ADAM automatic cell counter (NanoEnTek Inc, Seoul, South Korea) after plating.

### 4.4. RNA Preparation

Total RNA was isolated from cultured fibroblasts obtained from patients with SCI and healthy subjects using Trizol (Thermo Fisher Scientific, Waltham, MA, USA), according to the manufacturer’s instructions [32]. The quantity and purity of RNA were confirmed using a Nanodrop spectrophotometer (Thermo Fisher Scientific, Waltham, MA, USA). 

### 4.5. RNA Sequencing and Transcriptome Data Analysis 

TruSeqTM RNA Sample prep kits (Illumina, San Diego, CA, USA) were used to prepare the RNA-Seq transcriptome libraries from total RNA according to the manufacturer’s protocol [33]. Next, the library was sequenced with an Illumina HiSeq 2000plat form (Macrogen Corporation, Seoul, Korea). To check quality of RNA sequencing data, SolexaQA software [34] was used to investigate base quality scores from FASTQ files generated by Illumina sequencing technology. The reads from the FASTQ files were mapped against the human reference genome using TopHat version 2.0.6 (http://tophat.cbcb.umd.edu/). Transcripts with a fold change ≥ |2.0| and *p*-value < 0.05 were considered statistically significant and were included in downstream analysis.

### 4.6. Differentially Expressed Gene Identification and Pathway Analysis 

The lists of DEGs were compiled based on the following comparisons: fibroblasts from the upper limbs of SCI patients compared with healthy subjects and from the lower limbs of SCI patients compared with healthy subjects. DEGs were submitted to the Database for Annotation, Visualization, and Integrated Discovery (DAVID v6.7; http://david.abcc.ncifcrf.gov/) [35] via the Kyoto Encyclopedia of Genes and Genomes pathway analysis with a fold change ≥ |2.0| and *p*-value < 0.05. Among the numerous pathways identified, genes involved in the cell cycle pathway were validated.

### 4.7. Quantitative Real-Time Reverse Transcription Polymerase Chain Reaction

Quantitative real-time reverse transcription polymerase chain reaction (qRT-PCR) was conducted to validate the transcriptome analysis. Total RNA was reverse-transcribed into cDNA using a ReverTra Ace^®^ qPCR RT Master Mix with gDNA Remover (Toyobo, Osaka, Japan) according to the manufacturer’s instructions. The mRNA expression levels for genes of interest were profiled using qPCRBIO SyGreen Mix Hi-ROX (PCR BIOSYSTEMS, London, UK) with the StepOnePlus Real-Time PCR System (Applied Biosystems, Foster City, CA, USA). Data analysis was performed using the 2^−ΔΔCt^ method [36]. Primers used for qRT-PCR are described in Table S7.

### 4.8. Western Blot

Proteins were extracted from cultured fibroblasts obtained from patients with SCI and healthy subjects and were dissolved in RIPA buffer, boiled for 5 min, and loaded onto 4–12% Bis-Tris gels. Then, separated proteins were blotted onto polyvinylidene difluoride membranes (Invitrogen) with 20% (*v*/*v*) methanol in NuPage Transfer Buffer (Invitrogen) at 15 V for 4 h at 4 °C. The membranes were blocked for 1 h in tris-buffered saline containing 0.01% Tween 20 with 5% skim milk (Difco; BD Biosciences, Oxford, UK), then washed three times with tris-buffered saline containing 0.01% Tween 20 for 10 min. The blots were incubated overnight at 4 °C with the following primary antibodies specific to the target proteins: PLK1, CCNB2, CDC20 (1:1000; Abcam, Cambridge, England), CCNB1, BUB1, ERK, phosphorylated ERK, AKT, phosphorylated AKT, and GAPDH (1:1000; Santa Cruz Biotechnology, Santa Cruz, CA, USA). The next day, the blots were washed three times with TBST and incubated for 1 h with horseradish peroxidase-conjugated secondary antibodies (1:4000; Santa Cruz, CA, USA) at room temperature. The blots were washed three times with TBST, then visualized with an enhanced chemiluminescence detection system (Amersham Pharmacia Biotech, Little Chalfont, UK).

### 4.9. Statistical Analysis

All data are expressed as mean ± standard error of the mean. Statistical analyses were performed using the Statistical Package for Social Sciences version 25.0 (IBM Corp. Released 2015. IBM SPSS Statistics for Windows, Version 25.0. Armonk, NY: IBM Corp.). Variables between the groups were analyzed using one-way analysis of variance followed by the Bonferroni post hoc test. An independent *t*-test was used for the comparison between vehicle and treatment groups (tiotropium or acetylcholine) in SCI fibroblasts. A *p*-value < 0.05 was considered statistically significant. 

## Figures and Tables

**Table 1:** Enriched Kyoto Encyclopedia of Genes and Genomes pathways in the upper limbs of SCI patients.

Term	Count	*p*-Value	Genes	Fold Enrichment
**hsa04110:Cell cycle**	**30**	**5.46 × 10^−12^**	***E2F1***, ***TGFB3***, ***TTK***, ***PKMYT1***, ***PTTG1***, ***CCNE2***, ***CDC45***, ***MCM7***, ***CDKN2B***, ***BUB1***, ***CCNA2***, ***CDC6***, ***CDK1***, ***SMAD3***, ***CDC20***, ***MCM2***, ***MCM3***, ***CDC25C***, ***MCM4***, ***MCM5***, ***WEE1***, ***CDKN1C***, ***CCNB1***, ***CCND1***, ***CCNB2***, ***MAD2L1***, ***PLK1***, ***PCNA***, ***BUB1B***, ***GADD45B***	**4.503**
hsa04512:ECM-receptor interaction	17	6.22 × 10^−6^	*ITGA11*, *ITGA10*, *ITGB3*, *ITGA4*, *COL5A3*, *HMMR*, *LAMA4*, *CD36*, *COL6A6*, *CD44*, *COMP*, *ITGA7*, *COL1A2*, *RELN*, *COL1A1*, *THBS2*, *COL11A1*	3.797
hsa03030:DNA replication	11	1.12 × 10^−5^	*MCM7*, *LIG1*, *PRIM2*, *PCNA*, *POLA2*, *MCM2*, *MCM3*, *RNASEH2A*, *MCM4*, *FEN1*, *MCM5*	5.733
hsa04115:p53 signaling pathway	12	7.90 × 10^−4^	*CCNE2*, *CCNB1*, *CDK1*, *CCND1*, *CCNB2*, *CD82*, *RRM2*, *SERPINE1*, *PMAIP1*, *GADD45B*, *IGFBP3*, *GTSE1*	3.311
hsa04350:TGF-beta signaling pathway	13	0.002	*BMP4*, *NOG*, *LTBP1*, *CDKN2B*, *COMP*, *GDF5*, *TGFB3*, *SMAD3*, *ID4*, *SMURF2*, *ID3*, *THBS2*, *PITX2*	2.804
hsa04114:Oocyte meiosis	14	0.005	*CDK1*, *PKMYT1*, *AURKA*, *CDC20*, *IGF2*, *PTTG1*, *CDC25C*, *CCNE2*, *CCNB1*, *CCNB2*, *MAD2L1*, *PLK1*, *BUB1*, *FBXO5*	2.388
hsa05412:Arrhythmogenic right ventricular cardiomyopathy (ARVC)	11	0.006	*SLC8A1*, *SGCG*, *ITGA7*, *ITGA11*, *SGCD*, *ITGA10*, *ITGB3*, *ITGA4*, *CDH2*, *TCF7L2*, *SGCA*	2.716
hsa04510:Focal adhesion	20	0.010	*CAV1*, *PDGFA*, *ITGA11*, *ITGA10*, *ITGB3*, *ITGA4*, *COL5A3*, *CCND1*, *LAMA4*, *COL6A6*, *COMP*, *ITGA7*, *COL1A2*, *PDGFRA*, *RELN*, *PDGFD*, *COL1A1*, *THBS2*, *COL11A1*, *MYLK*	1.867
hsa05410:Hypertrophic cardiomyopathy (HCM)	11	0.014	*SLC8A1*, *ACTC1*, *SGCG*, *ITGA7*, *TGFB3*, *ITGA11*, *SGCD*, *ITGA10*, *ITGB3*, *ITGA4*, *SGCA*	2.428
hsa05414:Dilated cardiomyopathy	11	0.023	*SLC8A1*, *ACTC1*, *SGCG*, *ITGA7*, *TGFB3*, *ITGA11*, *SGCD*, *ITGA10*, *ITGB3*, *ITGA4*, *SGCA*	2.244
hsa05200:Pathways in cancer	27	0.024	*WNT5A*, *E2F1*, *FGFR2*, *FGF5*, *FGF7*, *PTGS2*, *PDGFA*, *TGFB3*, *KIT*, *TCF7L2*, *MMP1*, *CCNE2*, *WNT2*, *FOS*, *CDKN2B*, *RARB*, *HHIP*, *BMP4*, *FZD8*, *EPAS1*, *SMAD3*, *BIRC5*, *MECOM*, *STAT1*, *LAMA4*, *CCND1*, *PDGFRA*	1.545
hsa05416:Viral myocarditis	9	0.033	*ICAM1*, *CAV1*, *CCND1*, *SGCG*, *MYH11*, *SGCD*, *ITGB2*, *HLA-B*, *SGCA*	2.379
hsa04914:Progesterone-mediated oocyte maturation	10	0.038	*CCNB1*, *CDK1*, *MAD2L1*, *CCNB2*, *PLK1*, *BUB1*, *PKMYT1*, *IGF2*, *CDC25C*, *CCNA2*	2.182

Cell cycle pathway is relevant in the upper limbs of SCI patients and is shown in bold letters. These pathways are statistically significant (*p* < 0.05).

**Table 2:** Enriched Kyoto Encyclopedia of Genes and Genomes pathways in the lower limbs of SCI patients.

Term	Count	*p*-Value	Genes	Fold Enrichment
hsa04512:ECM-receptor interaction	12	0.000	*COL4A2*, *COL4A1*, *TNXB*, *TNXA*, *HMMR*, *LAMA1*, *SDC1*, *COMP*, *COL6A3*, *RELN*, *COL1A1*, *THBS2*, *COL11A1*	5.341
hsa04510:Focal adhesion	13	0.007	*CAV1*, *COL4A2*, *COL4A1*, *TNXB*, *TNXA*, *LAMA1*, *CCND1*, *COMP*, *COL6A3*, *RELN*, *PDGFD*, *COL1A1*, *COL11A1*, *THBS2*	2.418
hsa00480:Glutathione metabolism	6	0.010	*GSTM1*, *GGT5*, *GSTT2B*, *RRM2*, *ANPEP*, *GPX7*	4.487
**hsa04110:Cell cycle**	**9**	**0.017**	***CCNB1***, ***CCND1***, ***CCNB2***, ***PLK1***, ***BUB1***, ***BUB1B***, ***TTK***, ***CDC20***, ***PTTG1***	**2.692**
hsa04360:Axon guidance	9	0.021	*NRP1*, *UNC5B*, *SEMA3F*, *NTN4*, *NTNG1*, *SEMA3A*, *CXCL12*, *SLIT2*, *EPHA3*	2.609
hsa03320:PPAR signaling pathway	6	0.035	*OLR1*, *SCD*, *FABP3*, *SCD5*, *PCK2*, *ANGPTL4*	3.251
hsa00260:Glycine, serine and threonine metabolism	4	0.048	*PHGDH*, *DMGDH*, *PSAT1*, *CBS*	4.824
hsa00250:Alanine, aspartate and glutamate metabolism	4	0.048	*ASS1*, *ABAT*, *ASNS*, *GAD1*	4.824
hsa05020:Prion diseases	4	0.065	*EGR1*, *NCAM1*, *IL6*, *IL1B*	4.273
hsa04114:Oocyte meiosis	7	0.071	*CCNB1*, *CCNB2*, *PLK1*, *BUB1*, *CDC20*, *AURKA*, *PTTG1*	2.379

Cell cycle pathway is relevant in the lower limbs of SCI patients and is shown in bold letters. These pathways are statistically significant (*p* < 0.05).

**Table 3:** Common differentially expressed genes in the upper and lower limbs of SCI patients.

Common Genes	SCI-Upper vs. Healthy Control FC	SCI-Lower vs. Healthy Control FC
*CDC20*	7.532	3.483
*PTTG1*	6.591	2.591
*PLK1*	5.195	2.571
*CCNB2*	4.484	2.740
*CCNB1*	4.298	2.330
*BUB1B*	4.093	2.302
*BUB1*	3.722	2.155
*TTK*	3.410	2.130
*CCND1*	2.213	2.213

## Supplementary Materials

Supplementary materials can be found at https://www.mdpi.com/1422-0067/20/23/5982/s1.

## Abbreviations

SCI	Spinal cord injury
DEGs	Differentially expressed genes
FC	Fold change
CDC20	Cell division cycle 20
PLK1	Polo-like kinase 1
CCNB1	Cyclin B1
CCNB2	Cyclin B2
BUB1	BUB1 mitotic checkpoint serine/threonine kinase
