# Supplementary material for: Parasympathetic Effect Induces Cell Cycle Activation in Upper Limbs of Paraplegic Patients with Spinal Cord Injury
Source: Int J Mol Sci. 2019 Nov 27;20(23):5982. doi: 10.3390/ijms20235982 (PMC6929129; doi:10.3390/ijms20235982)
Supplement: Supplementary file 1 [file ijms-20-05982-s001.pdf]

International Journal of
*Molecular Sciences*

MDPI

**Supplementary Table S1.** Characteristics of patients with spinal cord injury.

| | **Subject 1** | **Subject 2** | **Subject 3** |
|---|---|---|---|
| Age | 57 | 49 | 59 |
| Gender | Male | Male | Male |
| Disease duration (months) | 58 | 73 | 15 |
| NLI | T5 | T4 | T3 |
| AIS | A | A | A |

NLI; Neurologic level of injury, AIS; American Spinal Injury Association impairment scale.

**Supplementary Table S2.** The gene expression ratios of SCI patients relative to healthy subjects.

| **Target Genes** | **SCI-Upper *vs.* Healthy Control. FC** | **SCI-Lower *vs.* Healthy Control. FC** |
|---|---|---|
| *CCNB1* | 2.81 ± 0.37 | 2.11 ± 0.58 |
| *CCNB2* | 4.49 ± 0.45 | 2.99 ± 0.76 |
| *PLK1* | 3.64 ± 0.38 | 2.44 ± 0.51 |
| *BUB1* | 6.19 ± 0.70 | 3.12 ± 0.81 |
| *CDC20* | 4.34 ± 0.50 | 3.08 ± 0.72 |

**Supplementary Table S3.** The protein expression ratios of SCI patients relative to healthy subjects.

| **Target Protein** | **SCI-Upper *vs.* Healthy Control. FC** | **SCI-Lower *vs.* Healthy Control. FC** |
|---|---|---|
| CCNB1 | 2.06 ± 0.19 | 1.20 ± 0.10 |
| CCNB2 | 4.31 ± 0.41 | 1.59 ± 0.06 |
| PLK1 | 8.80 ± 1.42 | 1.39 ± 0.17 |
| BUB1 | 3.38 ± 0.76 | 3.32 ± 0.54 |
| CDC20 | 5.12 ± 0.46 | 1.38 ± 0.08 |

**Supplementary Table S4.** The gene expression ratios in SCI patients treated with tiotropium and acetylcholine.

| **Target Genes** | **SCI-Upper+Tio *vs.* SCI-Upper+Veh. FC** | **SCI-Lower+Ach *vs.* SCI-Lower+Veh. FC** |
|---|---|---|
| *CCNB1* | 0.79 ± 0.03 | 1.93 ± 0.23 |
| *CCNB2* | 0.59 ± 0.09 | 1.62 ± 0.29 |
| *PLK1* | 0.23 ± 0.03 | 2.00 ± 0.27 |
| *BUB1* | 0.24 ± 0.02 | 1.75 ± 0.27 |
| *CDC20* | 0.56 ± 0.04 | 2.12 ± 0.08 |

**Supplementary Table S5.** The protein expression ratios in SCI patients treated with tiotropium and acetylcholine.

| Target Protein | SCI-Upper+Tio *vs.* SCI-Upper+Veh. FC | SCI-Lower+Ach *vs.* SCI-Lower+Veh. FC |
|---|---|---|
| CCNB1 | 0.78 ± 0.04 | 1.11 ± 0.07 |
| CCNB2 | 0.17 ± 0.02 | 1.14 ± 0.04 |
| PLK1 | 0.74 ± 0.06 | 1.24 ± 0.10 |
| BUB1 | 0.65 ± 0.05 | 2.20 ± 0.17 |
| CDC20 | 0.73 ± 0.03 | 1.51 ± 0.12 |

**Supplementary Table S6.** The protein expression ratios of cell proliferation in SCI patients treated with tiotropium and acetylcholine.

| Target Protein | SCI-Upper+Tio *vs.* SCI-Upper+Veh. FC | SCI-Lower+Ach *vs.* SCI-Lower+Veh. FC |
|---|---|---|
| Phosphorylated Erk /Total Erk | 0.75 ± 0.04 | 1.21 ± 0.07 |
| Phosphorylated Akt /Total Akt | 0.78 ± 0.05 | 1.33 ± 0.10 |

**Supplementary Table S7.** Primers used for qRT-PCR.

| Gene symbol | Forward primer (5′→3′) | Reverse primer (5′→3′) |
|---|---|---|
| ***CCNB1*** | **CTA CTG GGT CGG GAA GTC AC** | **AGC ATC TTC TTG GGC ACA CA** |
| ***CCNB2*** | **AAC AAG GCT ACA GCG TCG AA** | **CAA ATC ACT GGA CAC CGT CG** |
| ***PLK1*** | **TGA CTC AAC ACG CCT CAT CC** | **GCT CGC TCA TGT AAT TGC GG** |
| ***BUB1*** | **TCC GGC GGC TTC TAT TTG C** | **CCC ATT CAC CAA GAG GGT CAT** |
| ***CDC20*** | **AAT GTG TGG CCT AGT GCT CC** | **AGC ACA CAT TCC AGA TGC GA** |
| ***GAPDH*** | **AAG GGT CAT CAT CTC TGC CC** | **GTG AGT GCA TGG ACT GTG GT** |

*CCNB1*, Cyclin B1; *CCNB2*, Cyclin B2; *PLK1*, Polo-like kinase 4; *BUB1*, BUB1 mitotic checkpoint serine/threonine kinase; *CDC20*, cell division cycle 20; *GAPDH*, Glyceraldehyde 3-phosphate dehydrogenase.
